# Royal Ophthalmic Hospital

**Published:** 1902-05-17

**Authors:** 


### ROYAL OPHTHALMIC HOSPITAL.

### HEAVY AND INCREASING DEFICIT.

The year 1901 began with a deficiency of £2,591. The income during the year was £8,819, including £904 from legacies; the expenditure was £11,397, so that the excess of the latter was £2,578, making the total deficit at the end of the year £5,170. It was therefore hardly necessary to state that financially 1901 was a year of grave anxiety. Indeed, the liabilities to tradesmen became so heavy that the committee was compelled to seek a loan to enable them to keep the hospital open; the only alternative being either to curtail the scope of the work—already seriously restricted—or close the hospital altogether. After careful consideration the committee decided to transfer the Sedgwick Trust Fund to the Charity Commissioners, and ask them for an advance of £5,000. In order to repay this a portion of the trust money—£7,000—was separately invested, the interest on it being retained and reinvested by the Commissioners as a sinking fund. The hospital's income from dividends had of course by this been greatly reduced. Donations fell off from £4,386 in 1900 to £3,151, but subscriptions showed an encouraging rise of £200. Patients showed their appreciation of the work by increasing the amount collected in the hospital boxes by £16 to £462, and the Ladies' Guild helped to raise £322 by a ball.

